# An internal loop region is responsible for inherent target specificity of bacterial cold-shock proteins

**DOI:** 10.1261/rna.080163.124

**Published:** 2025-01

**Authors:** Satoshi Hasegawa, Rerina Inose, Mizuki Igarashi, Megumi Tsurumaki, Motofumi Saito, Tatsuo Yanagisawa, Akio Kanai, Teppei Morita

**Affiliations:** 1 Institute for Advanced Biosciences, Keio University, Tsuruoka, Yamagata 997-0017, Japan; 2 Faculty of Environment and Information Studies, Keio University, Fujisawa, Kanagawa 252-0882, Japan; 3 Graduate School of Media and Governance, Keio University, Fujisawa, Kanagawa 252-0882, Japan; 4 RIKEN Center for Biosystems Dynamics Research, Yokohama, Kanagawa 230-0045, Japan

**Keywords:** RNA-binding protein, cold-shock domain, bacteria, co-transcriptional regulation, small RNA

## Abstract

Cold-shock proteins (Csps), of around 70 amino acids, share a protein fold for the cold-shock domain (CSD) that contains RNA-binding motifs, RNP1 and RNP2, and constitute one family of bacterial RNA-binding proteins. Despite similar amino acid composition, Csps have been shown to individually possess inherent specific functions. Here, we identify the molecular differences in Csps that allow selective recognition of RNA targets. Using chimeras and mutants of *Escherichia coli* CspD and CspA, we demonstrate that Lys43-Ala44 in an internal loop of CspD, and the N-terminal portion with Lys4 of CspA, are important for determining their target specificities. Pull-down assays suggest that these distinct specificities reflect differences in the ability to act on the target RNAs rather than differences in binding to the RNA targets. A phylogenetic tree constructed from 1,573 Csps reveals that the Csps containing Lys-Ala in the loop form a monophyletic clade, and the members in this clade are shown to have target specificities similar to *E. coli* CspD. The phylogenetic tree also finds a small cluster of Csps containing Lys-Glu in the loop, and these exhibit a different specificity than *E. coli* CspD. Examination of this difference suggests a role of the loop of CspD-type proteins in recognition of specific targets. Additionally, each identified type of Csp shows a different distribution pattern among bacteria. Our findings provide a basis for subclassification of Csps based on target RNA specificity, which will be useful for understanding the functional specialization of Csps.

## INTRODUCTION

Posttranscriptional regulation of gene expression contributes to cellular processes in bacteria. This level of regulation is often completed with the aid of RNA-binding proteins (RBPs) (for review, see Holmqvist and Vogel 2018; Westermann 2018; Hör et al. 2020), including the conserved cold-shock proteins (Csps). These proteins, of around 70 amino acids, share a protein fold for the cold-shock domain (CSD) that contains RNA-binding motifs, RNP1 and RNP2, at which they bind RNA and single-stranded DNA (Schindelin et al. 1994; Jiang et al. 1997; Sachs et al. 2012). Widespread distribution of *csp* genes among bacteria suggests biological roles of Csps in fundamental processes. In *Escherichia coli*, of the nine Csp homologs (CspA to CspI), four, including CspA, increase upon cold shock, adjusting the gene expression pattern during the acclimation process from the cold-shock stress (Zhang et al. 2018). Some of the others have been reported to be expressed not in the cold-shock response but under some stringent conditions, such as minimal medium with a poor carbon source or long-term stationary phase (Yamanaka and Inouye 1997; Kram et al. 2020). Thus, because not all function under cold shock, the Csps are also referred to as CSD proteins.

Our current understanding of the function of Csps stems primarily from studies with the model organism *E. coli*, especially focusing on their activities to modulate transcription termination. Intrinsic termination is the major termination mechanism for bacterial transcription and is characterized by a terminator element that consists of a stem–loop followed by a U-rich tract in the nascent RNA (for review, see Ray-Soni et al. 2016; Chen et al. 2019). CspA, CspC, and CspE exhibited antitermination activities on several intrinsic terminators including the one within the *metY-rpsO* operon, presumably by preventing the formation of RNA secondary structure (Bae et al. 2000). Furthermore, recent studies suggested functional specialization of Csps. For instance, in *Salmonella*, in which six Csp homologs are present, CspC and CspE, but not other Csps, were shown to share RNA targets, and both protect one RNA target, *ecnB* mRNA encoding a bacteriolytic lipoprotein, from RNase E-dependent decay (Michaux et al. 2017). Additionally, CspC, but not CspE, alters the 5′ regulatory structure of *ugtL* mRNA encoding a virulence factor of *Salmonella*, resulting in translation activation (Choi et al. 2021). In *Staphylococcus aureus*, CspA, but not two other Csps, promotes the production of staphyloxanthin as well as some stress responses (Caballero et al. 2018; Catalan-Moreno et al. 2020). Therefore, each Csp seems to be individually assigned to cognate RNA targets, thereby serving their own functions, although they all possess conserved RNA-binding motifs.

CspD of *E. coli* is a member of the Csp family and the *cspD* gene is highly expressed in slow growth conditions such as minimal medium with a poor carbon source (Yamanaka and Inouye 1997; Morita et al. 2022). A previous study showed that CspD bound to RNA and single-stranded DNA and inhibited in vitro DNA replication of a plasmid with the *oriC* site; in vivo, overproduction of CspD caused a loss of cell viability (Yamanaka et al. 2001). Recently, our multicopy screen in *E. coli* identified CspD as an attenuator of intrinsic termination for a number of small regulatory RNAs (sRNAs) including SgrS sRNA (Morita et al. 2022). CspD binds to the target transcripts, attenuating the intrinsic termination for them and stabilizing the transcription products. An RNA-seq analysis further showed global effects of CspD, promoting transcription elongation across some regulatory RNA elements such as a riboswitch. Intriguingly, when the effects of overexpressing each of the *csp* genes from a plasmid were compared, the effect of CspD on SgrS termination was different from that of other Csps including CspA, and the *metY-rpsO* operon, a target of CspA, was little affected by CspD overexpression (Morita et al. 2022). These differences suggest that CspD could recognize specific RNA targets and/or act in a unique way on the nascent transcripts in comparison to other Csps. This has been reinforced by a recent global RNA interactome study, which demonstrated that CspD exhibited a different pattern of RNA/protein complexes than CspA, CspC, CspE, and CspG (Chihara et al. 2022). However, we have limited understanding of how the specificities of Csps are defined at the molecular level.

Here, we have constructed variants of *E. coli* CspD (*Ec*CspD) and CspA and compared their activities in order to identify the differences in Csps that allow selective recognition of RNA targets. Functional analyses demonstrate that Lys-Ala residues in an internal loop region and the N-terminal portion containing a Lys residue are important for determining target specificities of CspD and CspA, respectively. Phylogenetic analyses suggest that the identified residues can be effective in subclassification of bacterial Csps. Using the results of our work in conjunction with predicted structural models, how these Csps selectively act on cognate target RNAs is discussed.

## RESULTS

### Model target RNAs for assessing the specificity of Csps

In our previous study, differences in Csp specificity were found by comparing the effect on transcription termination for the gene encoding the SgrS small RNA in *E. coli*. To further investigate the target specificity of Csps, we used two chromosomal loci, the *baxL-bax* operon and the *gdx* gene, because our RNA-seq analysis demonstrated an increase in full-length transcripts from these genes under a CspD overproduction condition (Morita et al. 2022). The induction of *baxL-bax* mRNA was also observed during growth in M9 minimal medium with sodium acetate, a condition in which the chromosomal *cspD* gene is highly expressed.

Expression of both the *baxL-bax* operon and *gdx* has been reported to be cotranscriptionally regulated. The *baxL* gene encodes a small upstream ORF whose translation could modulate the expression of downstream *bax* encoding a putative glycoside hydrolase (Weaver et al. 2019). The expression of *gdx*, which encodes a small multidrug resistance (SMR) family transporter, is regulated by a guanidine II riboswitch in its upstream region (Sherlock et al. 2017; Kermani et al. 2020). Of note, a data set on transcription termination sites (TTS) from the Storz laboratory showed premature termination signals around these regions (Supplemental Fig. S1; Adams et al. 2021). This suggests that transcription elongation is likely modulated by these RNA elements, preventing the production of full-length mRNAs under normal growth conditions, which multicopy CspD can overcome.

**FIGURE 1.**
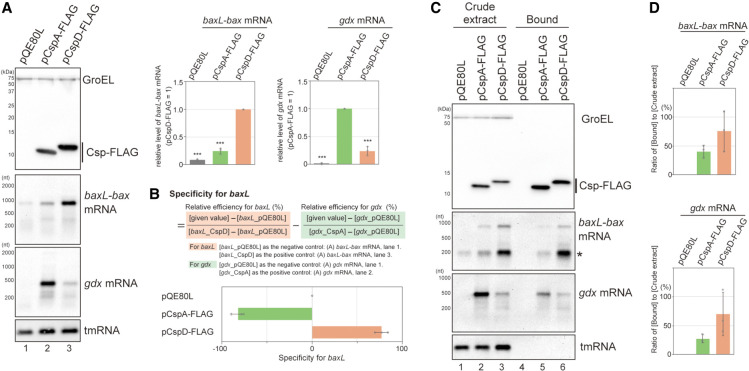
RNA elements that were used as model targets of Csps. (*A*) Properties of CspA and CspD. MG1655 cells harboring the indicated plasmids were grown in LB-ampicillin medium. At an OD_600_ of 0.3, 0.2 mM IPTG was added to cultures to induce Csps. Incubation was continued for 30 min. The protein sample was subjected to western blotting using anti-FLAG monoclonal antibody and anti-GroEL polyclonal antibodies. The RNA sample was subjected to northern blotting using probes for *baxL-bax* mRNA, *gdx* mRNA, or tmRNA. Relative RNA levels were calculated, with the RNA sample of CspD set to 1 for *baxL-bax* mRNA and CspA set to 1 for *gdx* mRNA. The results are averages of three independent experiments, with error bars representing the standard deviations. Statistical significance was calculated using an unpaired two-tailed Student's *t*-test. (***) *P* < 0.001. (*B*) Evaluation of target specificity. The specificity score was calculated by a formula in which the level of *gdx* mRNA relative to that seen for CspA (relative efficiency for *gdx*) was subtracted from the level of *baxL* mRNA relative to that seen for CspD (relative efficiency for *baxL*). The quantitative data in *A* were used to calculate each efficiency. The results are averages of three independent experiments, with error bars representing the standard deviations. (*C*) In vivo binding of CspA-FLAG and CspD-FLAG to *baxL-bax* mRNA and *gdx* mRNA. Crude extract was prepared from MG1655 cells harboring the indicated plasmids and subjected to the pull-down assay using anti-FLAG M2 magnetic beads, as described in Materials and Methods. (*D*) The ratio of (Bound) relative to (Crude extract) is shown; in the calculation for *baxL-bax* mRNA, the sum of the full-length and truncated (*) was used. The results are averages of three independent experiments, with error bars representing the standard deviations.

Since the expression patterns of *E. coli csp* genes differ, we employed a pQE80L plasmid system in which a cloned gene is ectopically overexpressed under the control of an IPTG inducible promoter. *E. coli cspA* and *cspD* genes were individually cloned into the vector. The 5′ untranslated sequence originated from the vector; for protein analyses, a *FLAG* sequence was inserted just upstream of the stop codon. Cells harboring the vector or each pQE-Csp-FLAG were cultivated to early exponential phase, and, to induce the *csp-FLAG* gene, the cultivation was continued for 30 min in the presence of 0.2 mM IPTG. Western blot analysis showed that FLAG-tagged CspA and CspD proteins were produced to nearly identical levels (Fig. 1A). To measure the protein level of CspD-FLAG, we compared it with chromosomally encoded CspD-FLAG in M9 minimal medium with sodium acetate in which the endogenous *cspD* gene was highly expressed (Morita et al. 2022). Western blotting estimated the plasmid-borne CspD-FLAG was accumulated around 40 times more than the chromosomally encoded CspD-FLAG (Supplemental Fig. S2A). We however adopted the approach with the pQE-system because it allows comparisons of Csps under the same growth condition.

Northern blot analyses with probes for the 5′ region of *baxL* or *gdx* (Supplemental Fig. S1) demonstrated that in the vector control strain full-length transcripts of *baxL-bax* mRNA and *gdx* mRNA were detected at low levels; these mRNAs increased in cells overexpressing CspD (Fig. 1A, lanes 1 and 3), consistent with what was seen in the previous RNA-seq analysis. In comparison with the CspD overproduction, CspA exhibited a moderate effect on *baxL-bax* expression and a pronounced effect in increasing full-length *gdx* mRNA (Fig. 1A, lane 2). This difference was confirmed in cells overproducing nontagged proteins (Supplemental Fig. S2B).

We evaluated the target specificity of these Csps with a formula in which the level of *gdx* mRNA relative to that seen for cells overexpressing CspA (relative efficiency for *gdx*) was subtracted from the level of *baxL* mRNA relative to that seen for CspD (relative efficiency for *baxL*) (Fig. 1B). This comparison emphasizes the very different specificity of CspD compared to that of CspA. These results indicate that CspD and CspA can be divided based on both the induction levels of these two mRNAs and target specificity; CspD preferentially supports the *baxL-bax* regulation, while CspA has a stronger effect on the *gdx* riboswitch.

We then tested if CspA and CspD bind to the target mRNAs by in vivo pull-down assay with anti-FLAG M2 magnetic beads. The *baxL-bax* and *gdx* mRNAs, but not transfer-messenger RNA (tmRNA), were copurified with CspD-FLAG and CspA-FLAG (Fig. 1C), suggesting that CspD and CspA directly act on the *baxL* and *gdx* RNAs. For the *baxL-bax* mRNA, its band in extracts was considerably shorter than seen in Figure 1A (compare size of band with asterisk in 1C to major band in 1A). It seems likely that this smaller band is protected from degradation in the extract, possibly by binding to the Csps. We also note that, although differences were observed in the amount of copurified mRNAs, enrichment ratios for these bands (bound/total) were comparable for CspA and for CspD for each of the mRNA targets (Fig. 1D). Together, these suggest that the differences in Csp specificity do not reflect different abilities to bind these sequences but instead the ability to promote accumulation of these RNA bands before cells were broken open.

### Regions involved in specific actions of CspD and CspA

**FIGURE 2.**
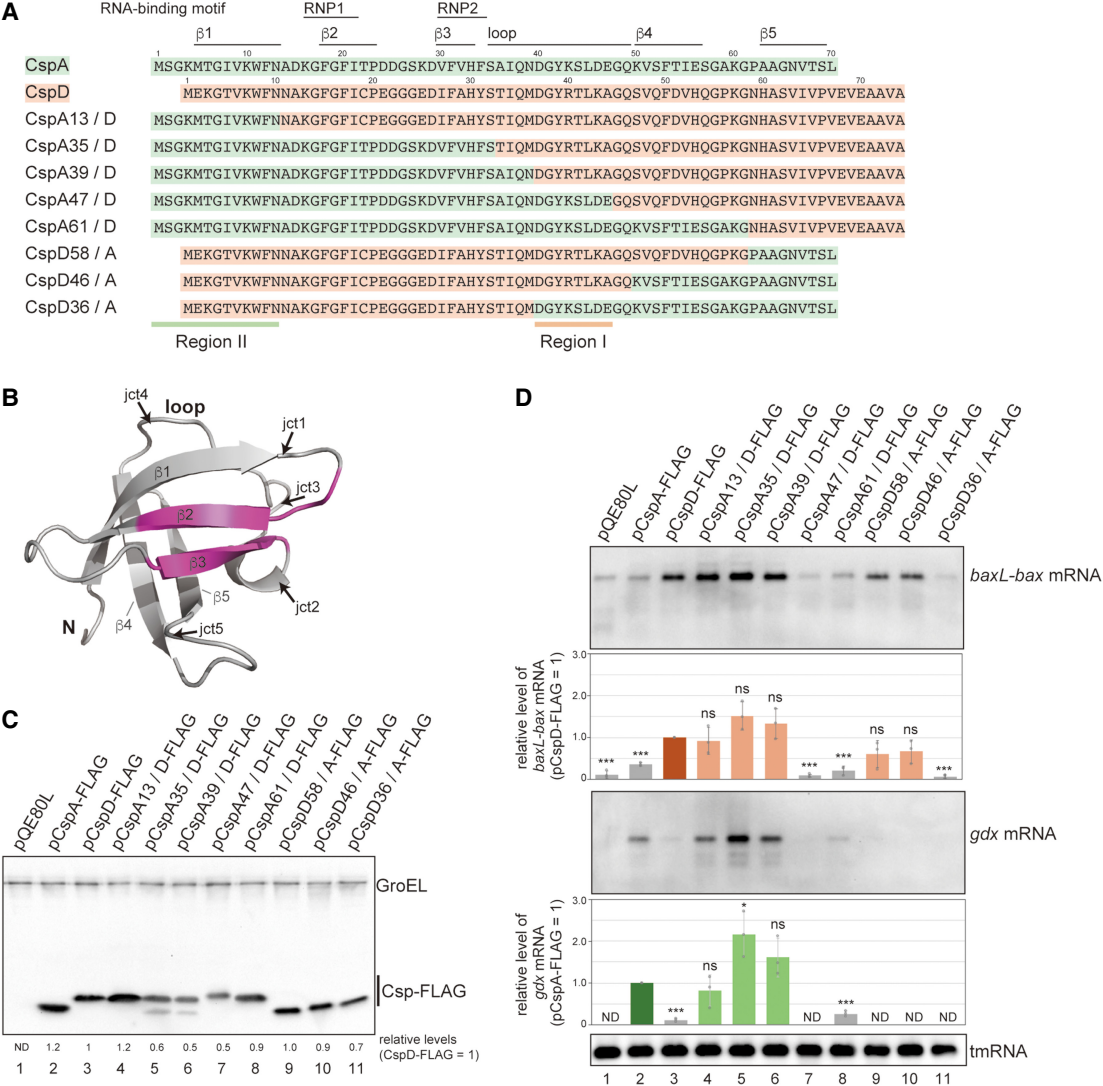
Chimeric variants of CspD and CspA. (*A*) Sequence alignment of CspA, CspD, and chimeras. For chimeras, the regions originating from CspA and CspD are marked in green and orange, respectively. RNA-binding motifs, RNP1 and RNP2, a loop region, and β-strands are indicated by lines on *top* of the sequence. (*B*) The structure of *E. coli* CspA monomer (Protein Data Bank ID: 1MJC). The junctions in chimeras (jct1 to jct5) are indicated by arrows. (*C*,*D*) Expression and properties of chimeras. MG1655 cells harboring the indicated plasmids were grown in LB-ampicillin medium. At an OD_600_ of 0.3, 0.2 mM IPTG was added to cultures to induce Csps. Incubation was continued for 30 min. (*C*) The protein sample was subjected to western blotting using anti-FLAG monoclonal antibody and anti-GroEL polyclonal antibodies. (*D*) The RNA sample was subjected to northern blotting using probes for *baxL-bax* mRNA, *gdx* mRNA, or tmRNA. Quantitation data are shown *below* the northern blot; averages of relative RNA levels were calculated from three independent experiments, with error bars representing the standard deviations. The RNA samples of CspD and CspA were set to 1 for *baxL-bax* mRNA and *gdx* mRNA, respectively. Statistical significance was calculated using an unpaired two-tailed Student's *t*-test. (ns) Not significant, (*) *P* < 0.05, (***) *P* < 0.001.

To identify the regions that dictate the specific target recognition by CspD and CspA, we generated genes encoding chimeric proteins in the pQE80L expression plasmid. As shown in Figure 2A, the chimeras were designed as either CspA/D, in which the N-terminal 13, 35, 39, 47, or 61 residues of CspA were fused to the C-terminal corresponding positions of CspD, or CspD/A, in which the N-terminal 58, 46, or 36 residues of CspD were fused to the C-terminal corresponding positions of CspA. Each junction site is designed to be around the borders of structural elements: after the β-strand 1, after β-strand 3, within a loop, before β-strand 4, and before β-strand 5 (Fig. 2A,B). Western blot analysis showed that all chimeric proteins were successfully produced (Fig. 2C). Since intracellular levels of proteins may somehow affect the target induction by Csps, the relative ratios between full-length proteins were indicated on the bottom.

Full-length *baxL-bax* and *gdx* mRNAs were detected by northern blotting, followed by quantitation. Overproduction of CspA13/D, CspA35/D, CspA39/D, CspD58/A, and CspD46/A led to the induction of *baxL-bax* mRNA similarly to what was seen with the wild-type CspD (Fig. 2D, compare lane 3 to lanes 4, 5, 6, 9, 10), whereas CspA47/D, CspA61/D, and CspD36/A did not result in *baxL-bax* mRNA expression (Fig. 2D, lanes 7, 8, 11). The chimeric proteins that act on the *baxL-bax* mRNA contain a portion of a loop in CspD; those that did not act on *baxL* lacked this portion, revealing that this region is necessary for the *baxL* recognition (defined as Region I from CspD in Fig. 2A).

*gdx* mRNA was high in the presence of the CspA plasmid; overproduction of CspA13/D, CspA35/D, and CspA39/D all had effects on *gdx* similar to that of CspA (Fig. 2D, compare lane 2 to lanes 4–6). These proteins contain the N-terminal portion of CspA, suggesting that this region is involved in *gdx* recognition (defined as Region II from CspA in Fig. 2A). CspA47/D and CspA61/D also contain Region II, but they did not induce the *gdx* mRNA possibly due to disfunction of these fusion junctions (Fig. 2D, lanes 7, 8). It is worth noting that CspA13/D, CspA35/D, and CspA39/D exhibited an ability to recognize both *baxL-bax* mRNA and *gdx* mRNA (Fig. 2D, lanes 4–6). These suggest that the ways in which CspD and CspA recognize target RNAs are not mutually exclusive.

### Dissection of Regions I and II

**FIGURE 3.**
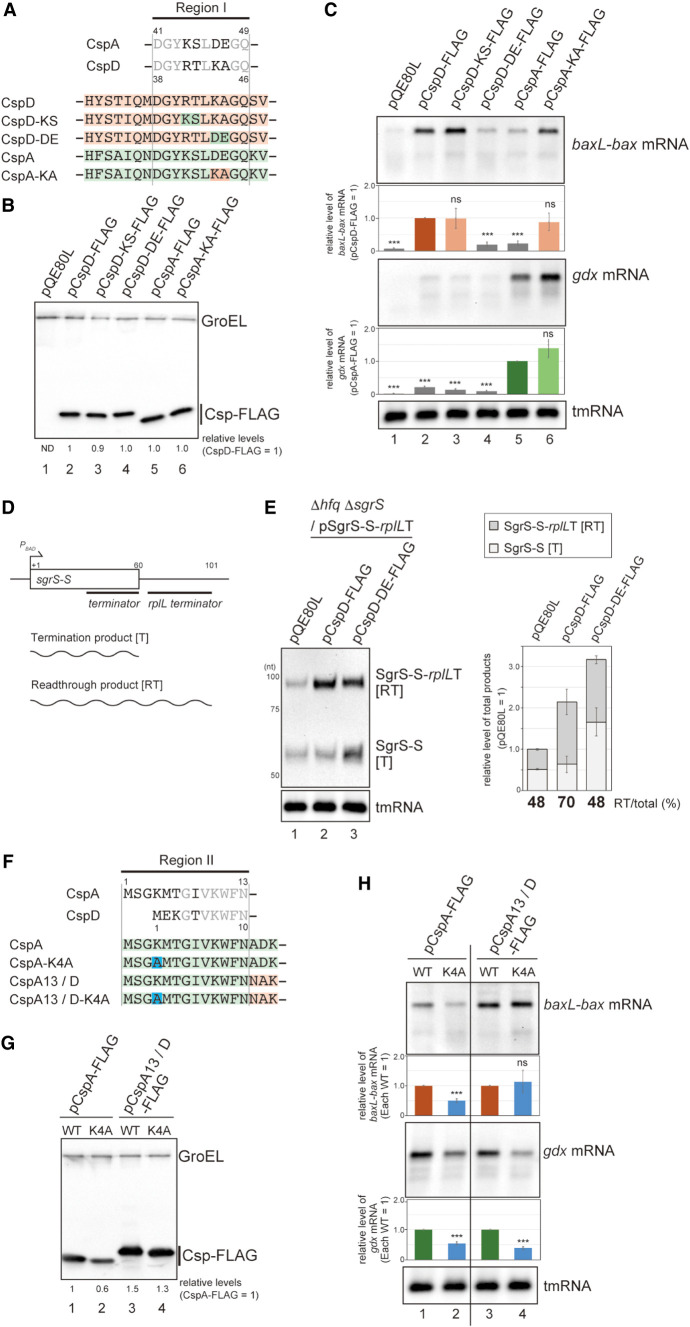
Dissection of Region I and Region II. (*A*) Sequence alignment of Region I. Conserved residues between CspA and CspD are shown in gray. The regions originating from CspA and CspD are marked in green and orange, respectively, and mutation sites are indicated in the colors of the originating Csp. (*B*,*C*) Effect of mutations in Region I. MG1655 cells harboring the indicated plasmids were grown in LB-ampicillin medium. At an OD_600_ of 0.3, 0.2 mM IPTG was added to cultures to induce Csps. Incubation was continued for 30 min. (*B*) The protein sample was subjected to western blotting using anti-FLAG monoclonal antibody and anti-GroEL polyclonal antibodies. (*C*) The RNA sample was subjected to northern blotting using probes for *baxL-bax* mRNA, *gdx* mRNA, or tmRNA. Quantitation data are shown *below* the northern blot; averages of relative RNA levels were calculated from three independent experiments, with error bars representing the standard deviations. The RNA samples of CspD and CspA were set to 1 for *baxL-bax* mRNA and *gdx* mRNA, respectively. (*D*,*E*) A double terminator system for SgrS (Morita et al. 2015), and effect of a mutant CspD. TM772 (Δ*hfq* Δ*sgrR-sgrS*) cells harboring the indicated plasmids were grown in LB kanamycin, ampicillin medium. At an OD_600_ of 0.2, 0.2 mM IPTG was added to cultures to induce Csps, and incubation was continued for 60 min. Then, 0.4% arabinose was added to cultures to induce *sgrS-S*-*rplL*T, and incubation was continued for 10 min. The RNA sample was subjected to northern blotting using probes for SgrS-S or tmRNA. Quantitation of the northern blot data is shown to the *right*. *Upper* dark gray bars indicate the readthrough transcript (RT); *lower* light gray bars indicate the terminated product (T), the total bar indicates the sum of T and RT. Relative RNA levels are calculated, with the RNA sample of the vector control set to 1. Percentage of the RT relative to total is shown at the *bottom*. (*F*) Sequence alignment of Region II. Conserved residues between CspA and CspD are shown in gray. The regions originating from CspA and CspD are marked in green and orange, respectively, and mutation sites are indicated in blue. (*G*,*H*) Effect of mutations in Region II. MG1655 cells harboring the indicated plasmids were analyzed as in *B*. Quantitation data are shown *below* the northern blot; averages of relative RNA levels were calculated from three independent experiments, with error bars representing the standard deviations. The RNA samples of each parental plasmid were set to 1. Statistical significance was calculated using an unpaired two-tailed Student's *t*-test. (ns) Not significant, (***) *P* < 0.001.

To further study the recognition of *baxL* by the Csps, Region I was dissected. Only four amino acid residues differ between CspD and CspA in this region (Fig. 3A). We generated CspD mutants in which these residues of CspD were substituted with those of CspA (Fig. 3A,B). Replacing 43K-44A with D-E weakened the induction of full-length *baxL-bax* mRNA to a level comparable to CspA, whereas the replacement of 41R-42T with K-S did not alter the level of *baxL* induction (Fig. 3C, lanes 3 and 4). A mutant CspA in which D46-E47 were replaced with K-A consistently increased *baxL-bax* mRNA, making it comparable to CspD (Fig. 3C, lane 6). These results indicate that the two residues at position 43-44 of CspD determine the ability of a Csp to act on *baxL*. For *gdx* mRNA, the exchange of K-A and D-E showed no overt effect (Fig. 3C), supporting the result of the chimeric experiments that *gdx* can be recognized at Region II in an independent manner from Region I.

The role of CspD as an attenuator of transcription termination was derived from its identification in our genetic screen for bypassing the terminator of SgrS sRNA. We examined the effect of the CspD-DE mutant on SgrS termination. For the analysis, we employed a plasmid system in which the stable intrinsic terminator of *rplL* was fused to the minimal functional region of SgrS (denoted as SgrS-S; Fig. 3D) (Morita et al. 2015, 2022). To exclude the Hfq-dependent stabilization of sRNAs, the experiment was conducted in a Δ*hfq* background. In the vector control strain, both SgrS-S (T) and its readthrough product (RT) were detected at comparable levels, and CspD overproduction increased the level of readthrough product, and therefore the ratio of RT to total (Fig. 3E), confirming the attenuator function of CspD. In contrast, overproduction of CspD-DE no longer altered the ratio of readthrough product, indicating that the K-A residues in the loop are necessary for the CspD action to attenuate SgrS termination. Note that the level of each product was increased by the overproduction of CspD-DE (Fig. 3E, compare lane 3 to lane 1). We previously showed that, in addition to its role as an attenuator of termination, CspD stabilized both SgrS-S and the readthrough product (Morita et al. 2022). Thus, the increase in these transcripts by CspD-DE suggests that the K-A residues do not affect the stabilizer role.

The effect of CspA on *gdx* depended on Region II (see Fig. 2). The Region II in CspA contains an unstructured extension at the N-terminus, which is not present in CspD (Fig. 3F). We focused on a basic residue K4 in Region II and tested its role in *gdx* recognition (Fig. 3F,G). For CspA and chimeric protein CspA13/D, both of which induced *gdx* mRNA, the replacement of K4 with alanine somewhat diminished the induction level (Fig. 3H, lanes 2 and 4). These indicate that K4 in CspA is important for the full activity of these Csps on *gdx*. For *baxL*, the level of full-length mRNA was less elevated by CspA overproduction than by CspA13/D, since CspA does not contain an active K-A in Region I (Fig. 3H, lanes 1 and 3, and see Fig. 2). This weak induction relied in part on K4 because the alanine replacement further reduced the level of *baxL-bax* mRNA (Fig. 3H, lane 2). In contrast, CspA13/D-K4A, which contains an active Region I derived from CspD, was unaffected for induction of *baxL-bax* mRNA compared to CspA13/D (Fig. 3H, lane 4). These results confirm, as shown above, that Region I of CspD is the primary region to recognize *baxL* and suggest that, in the absence of the K-A in Region I, Region II can partially substitute, as in CspA.

### Acidic residues in the loop impede *baxL* induction

**FIGURE 4.**
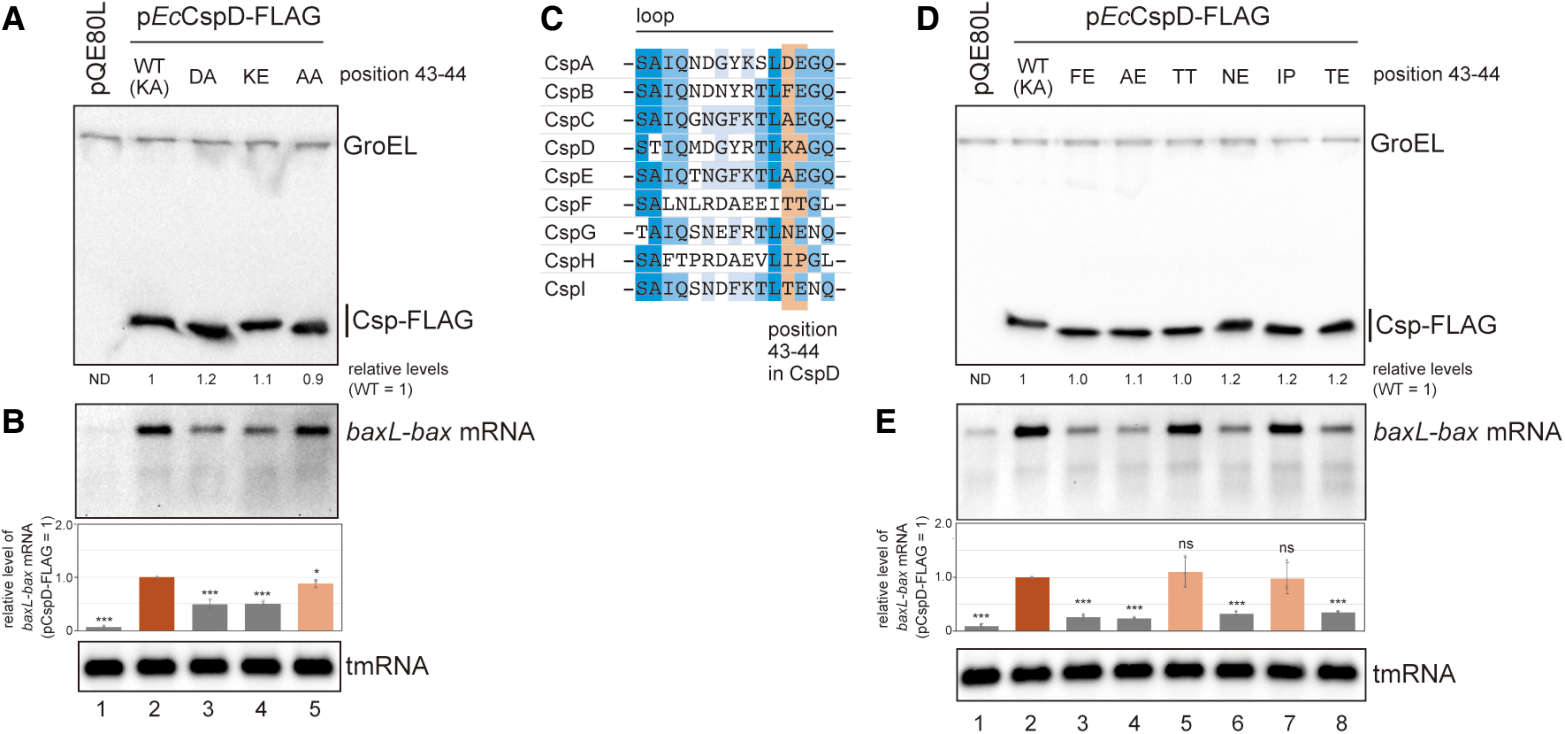
Role of the CspD loop region. (*A*,*B*) Point mutations in position 43-44 of CspD. MG1655 cells harboring the indicated plasmids were grown in LB-ampicillin medium. At an OD_600_ of 0.3, 0.2 mM IPTG was added to cultures to induce Csps. Incubation was continued for 30 min. (*A*) The protein sample was subjected to western blotting using anti-FLAG monoclonal antibody and anti-GroEL polyclonal antibodies. (*B*) The RNA sample was subjected to northern blotting using probes for *baxL-bax* mRNA or tmRNA. Quantitation data are shown *below* the northern blot; averages of relative RNA levels were calculated from three independent experiments, with error bars representing the standard deviations. The RNA sample of CspD was set to 1. (*C*) Sequence alignment of the loop region in *E. coli* Csps. Amino acid residues with more than 80% identity across all of the shown Csps are marked in dark blue; those with identities of 60%–80% and 40%–60% are marked in blue and light blue, respectively. The CspD 43-44 positions are highlighted in orange. (*D*,*E*) Exchange of two amino acid residues of the CspD loop with those of other *E. coli* Csps. MG1655 cells harboring the indicated plasmids were analyzed as in *A*. Statistical significance was calculated using an unpaired two-tailed Student's *t*-test. (ns) Not significant, (*) *P* < 0.05, (***) *P* < 0.001.

Two amino acid residues in the loop were found to determine the *baxL* induction by Csps (Fig. 3C). Composition of these residues in CspD is a basic residue K43 followed by A44; the corresponding positions in CspA are composed of acidic residues D46 and E47. To clarify whether the induction of *baxL-bax* mRNA is caused by the positive charge of a basic residue or is diminished by the negative charge of acidic residues, we investigated CspD mutants in which K-A were individually replaced with various residues (Fig. 4A). In the cells overproducing mutants CpsD-DA or CspD-KE, resulting from single substitutions K43D or A44E, the *baxL-bax* mRNA was less induced, whereas overproduction of CspD-AA mutant led to the *baxL* induction comparable to the wild-type (Fig. 4B). To extend the investigation, we looked at corresponding positions in the loop region of the seven other Csps of *E. coli*. The positions to K43-A44 of CspD vary in each of these proteins; these are composed of F-E, A-E, T-T, N-E, I-P, and T-E in CspB, CspC/CspE, CspF, CspG, CspH, and CspI, respectively (Fig. 4C; Supplemental Fig. S3A). We generated the CspD mutants in which the K-A was replaced with each of these sets of amino acid residues (Fig. 4D). The replacement of KA to TT or IP retained the induction of *baxL-bax* mRNA while the mutants having X-E residues in the loop reduced its induction (Fig. 4E). Taken together, these results indicate that acidic residues in the loop negatively act on target recognition or action. A detailed mechanism based on the structure models is discussed below.

We additionally examined the properties of seven *E. coli* Csps. Western blot analysis showed that the FLAG-tagged CspA, CspC, CspD, CspE, CspF, CspG, CspH, and CspI proteins were produced to nearly identical levels (Supplemental Fig. S3B). CspB-FLAG accumulated less (Supplemental Fig. S3B, lane 3), presumably due to either reduced stability of this protein or inefficient translation under this growth condition.

Northern blot analysis was used to compare the effect of overproducing the other Csps to the effects of overproducing CspA or CspD (strong induction of *gdx* mRNA or *baxL-bax* mRNA, respectively) (Supplemental Fig. S3C, lanes 2 and 5). The overexpression of CspC, CspE, CspG, and CspI exhibited similar effects to CpsA; *baxL-bax* mRNA was moderately induced and the *gdx* mRNA was markedly increased (Supplemental Fig. S3C, lanes 4, 6, 8, and 10). In good agreement with the results of our assays with chimeric proteins, these four Csps possess the expected features: K4 in Region II and negatively charged residues in Region I, as CspA does. In the cells overexpressing CspB, CspF, and CspH, neither mRNA was much affected (Supplemental Fig. S3C, lanes 3, 7, and 9). CspB is poorly expressed (Supplemental Fig. S3B), and CspF and CspH exhibit low identities to CspA and CspD (Supplemental Fig. S3A). These features may explain the lower function on both *baxL-bax* and *gdx* mRNAs, but further work is needed to identify regions that distinguish CspB, CspF, and CspH from CspA and CspD.

### Phylogenetic analysis of Csps focused on loop and N-terminal specificity residues

**FIGURE 5.**
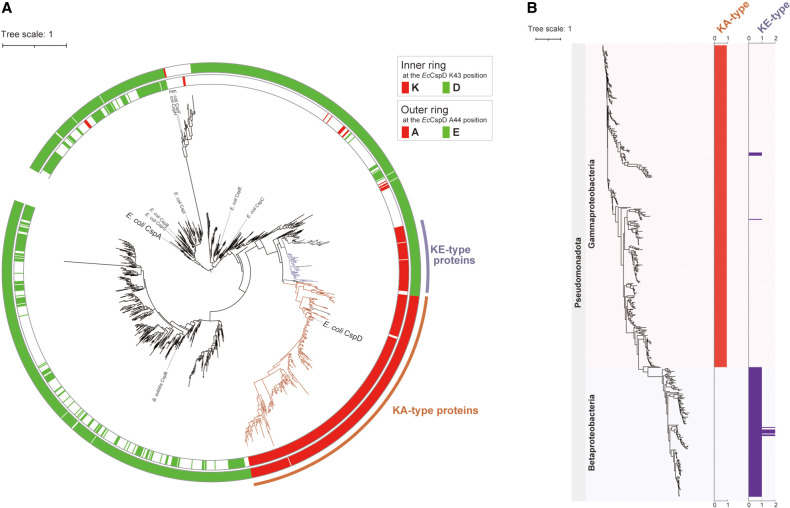
(*A*) A phylogenetic tree composed of 1573 Csps, visualizing amino acid residues at certain positions. The detailed method is described in Materials and Methods. The Csps that were used as queries for the PSI-BLAST search are shown; two *Salmonella* Csps (GCF_000210855.2: CBW17860.1, CBW16717.1) were not indicated because they were not included in the RefSeq database (October 2022 data set). Inner ring: proteins that contain K or D at the same position as *Ec*CspD K43 are represented by red or green, respectively. Outer ring: proteins that contain A or E at the same position as *Ec*CspD A44 are represented by red or green, respectively. Clusters of K-A and K-E types are indicated in orange and purple, respectively. (*B*) Distribution of K-A and K-E types in bacterial genomes. A phylogenetic tree was constructed from the bacterial genomes, in which one or more genes encoding the K-A type and K-E type are present. Each type of Csp was mapped to the owner genomes. Bacterial phyla and classes are shown to the *left*. *Horizontal* axes represent the number of each type of the contained Csps. The detailed method is described in Materials and Methods.

Although members of the Csp family have been bioinformatically annotated, it is still difficult to define their specific functions. In the chimera and site-directed mutant experiments so far, K-A residues in the loop of *Ec*CspD were demonstrated to ensure its target selectivity. We suspected that these residues would allow us to predict the *Ec*CspD-like target specificity across bacteria. A large-scale similarity search using PSI-BLAST (see Materials and Methods) detected 2,440 CDSs of Csps from 1,019 bacterial genome (Supplemental Table S1). To create a phylogenetic tree of these Csps, duplicate identical CDSs were excluded, resulting in 1,573 CDSs. We then asked what amino acid residue is present at the K43-A44 position of *Ec*CspD in the multiple sequence alignments. A lysine residue at the same position as K43 of *Ec*CspD was found in 427 proteins (red on inner ring of Fig. 5A), while an aspartate residue at the same position as D46 of *Ec*CspA was found in 327 proteins (Fig. 5A, green on inner ring). An alanine residue at the same position as A44 of *Ec*CspD was found in 325 proteins (red on outer ring of Fig. 5A), while a glutamate residue at the same position as E47 of *Ec*CspA was found in 1,205 proteins (Fig. 5A, green on outer ring). Of note, the proteins including *Ec*CspD that contain K-A in the loop formed a monophyletic clade (Fig. 5A, referred to as K-A type proteins). This infers that these K-A residues are effective in defining the Csp that functions like *Ec*CspD.

The phylogenetic tree additionally found a small cluster that is composed of proteins containing K-E residues in the loop (Fig. 5A, referred to as K-E type proteins). In others, the first position seems to be variable, although most of the second position is dominated by a glutamate residue. For the K-X proteins, the position corresponding to X is dominated almost exclusively by A (320 CDSs) or E (102 CDSs) in 98.8% of the total (427 CDSs).

We also selected the proteins that contain a lysine residue at the same position as K4 of *Ec*CspA and compared those with K-A and K-E type proteins according to the K43 of *Ec*CspD. Most of the K4 type proteins were detected separately from K43 type proteins, with only 13 of 1,573 proteins containing both lysine residues (Supplemental Fig. S4, indicated with blue on ring). These results suggest that the lysine residue in the N-terminal unstructured portion may predict Csps that function like *Ec*CspA. We note, however, that the lysine residue at this position is conserved even in CspB, CspF, and CspH of *E. coli*, which either accumulated to lower levels or showed little induction of both *baxL-bax* mRNA and *gdx* mRNA (see Supplemental Fig. S3). Therefore, K4 is not sufficient for CspA-like function.

### Distribution of each type of Csp in bacterial genomes

We then studied the phylogenetic distribution of Csps. A phylogenetic tree was generated from 4,031 bacterial genomes that we used for our large-scale similarity search, and the included Csps were connected to the owner bacterium (Supplemental Fig. S5A). Extended branches on the tree indicate the number of *csp* genes per bacterial genome. The presence and absence of the Csps that were obtained with our PSI-BLAST search appear to be constrained by phylum; they were detected extensively in Pseudomonadota (Gamma- and Beta- but not Alphaproteobacteria) and Bacillota, and in parts of Actinomycetota and others. We note that, as our similarity search was performed to detect very similar members to queries including *Ec*CspD (see Materials and Methods), subfamilies containing more divergent CSDs may have been excluded. A recent study on RNA regulation in *Bacteroides* found a gene encoding a CSD protein BT_1884 and showed that it complemented some phenotypes of mutated *Salmonella* lacking genes *cspC* and *cspE* (Prezza et al. 2022). This *Bacteroides* CSD protein had a low identity to the Csps used as queries in this study because it contains a long extension of about 80 amino acids at the N-terminus and was not detected as a candidate with our search.

The number of Csps ranges from one to ten and does not seem to be strongly constrained by phylum (Supplemental Fig. S5A). We were interested in the relationship between the number of Csps and the characteristics of the owner genome. By comparing the group with three or more Csps to that with one or two, the mean genome size and GC-content in the former group were statistically larger and higher, respectively, and less varied (Supplemental Fig. S5B). This might reflect the usefulness of Csps in regulating transcripts that tend to form stable structures due to the high GC-content and on large genomes.

The included Csps were highlighted by types of K-A and K-E (Fig. 5B, also listed in Supplemental Table S1). The K-A type proteins, including *Ec*CspD, exhibited widespread distribution in Gammaproteobacteria, while the K-E type was mainly found in Betaproteobacteria. Each genome generally encoded only one K-A type or one K-E type, with a few exceptions. Taken together with the tree of 4031 bacterial genomes (Supplemental Fig. S5A), these suggest that Csps of other phyla such as Bacillota were solely built from those outside these identified types. These differences in distribution infer that each type of Csps plays an inherent role in bacterial physiology for diverse habitats. Specifically, given both the distinct clusters of the K-A and K-E type on the molecular phylogenetic tree and the constrained distribution of each type among Gamma- and Betaproteobacteria, it could be imagined that these types likely evolved independently of other Csps as monophyletic lineages after their divergence from a common ancestor.

### Target specificities of Csps from other bacteria

**FIGURE 6.**
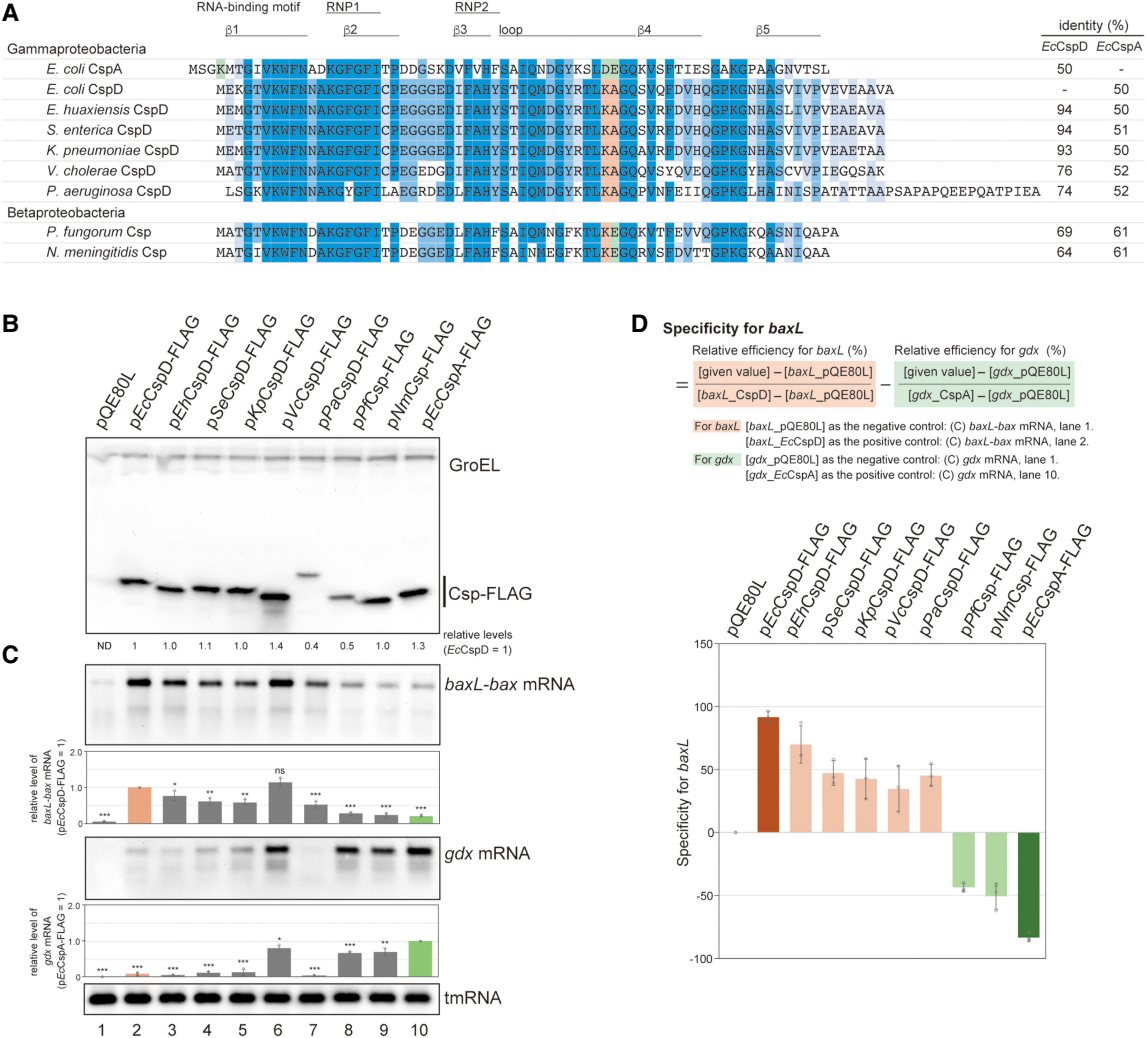
Csps grouped by identified amino acid residues. (*A*) Sequence alignment of the Csps tested. K4, D46, and E47 in *Ec*CspA and the identical residues at the same position are highlighted in green, whereas K43 and A44 in *Ec*CspD and the identical residues at the same position are highlighted in orange. Amino acid residues with more than 80% identity across all of the shown Csps are marked in dark blue; those with identities of 60%–80% and 40%–60% are marked in blue and light blue, respectively. Percent identities of Csps with *Ec*CspD and *Ec*CspA based on comparison by the NCBI BLAST are indicated to the *right*. (*B*,*C*) Expression and properties of the grouped Csps. MG1655 cells harboring the indicated plasmids were grown in LB-ampicillin medium. At an OD_600_ of 0.3, 0.2 mM IPTG was added to cultures to induce Csps. Incubation was continued for 30 min. (*B*) The protein sample was subjected to western blotting using anti-FLAG monoclonal antibody and anti-GroEL polyclonal antibodies. (*C*) The RNA sample was subjected to northern blotting using probes for *baxL-bax* mRNA, *gdx* mRNA, or tmRNA. Quantitation data are shown *below* the northern blot; averages of relative RNA levels were calculated from three independent experiments, with error bars representing the standard deviations. The RNA samples of *Ec*CspD and *Ec*CspA were set to 1 for *baxL-bax* mRNA and *gdx* mRNA, respectively. Statistical significance was calculated using an unpaired two-tailed Student's *t*-test. (ns) Not significant, (*) *P* < 0.05, (**) *P* < 0.01, (***) *P* < 0.001. (*D*) Evaluation of target specificity. The specificity score was calculated by a formula in which the level of *gdx* mRNA relative to that seen for CspA (relative efficiency for *gdx*) was subtracted from the level of *baxL* mRNA relative to that seen for CspD (relative efficiency for *baxL*). The quantitative data in *C* was used to calculate each efficiency. The results are averages of three independent experiments, with error bars representing the standard deviations.

To confirm the target specificity of the K-A type and K-E type, we experimentally investigated seven selected proteins (Fig. 6). Five of the K-A type proteins were manually selected, which had been annotated as CspD on the NCBI database likely based on sequence similarity (*Enterobacter huaxiensis*, *Salmonella enterica*, *Klebsiella pneumoniae*, *Vibrio cholerae,* and *Pseudomonas aeruginosa*). Two of the K-E type proteins were also selected (*Paraburkholderia fungorum* and *Neisseria meningitidis*). The percentage identities of these Csps to *Ec*CpsD ranged from 94% to 64%; those to *Ec*CspA ranged from 61% to 50% (Fig. 6A).

Each of the seven selected proteins was produced from a pQE-plasmid in *E. coli* (Fig. 6B). The specificity of each protein was evaluated by comparing levels of *baxL-bax* and *gdx* mRNAs. In cells expressing K-A type proteins, *baxL-bax* mRNA was induced at comparable levels to the control *Ec*CspD (Fig. 6C, compare lanes 3–7 with lane 2). This induction of *baxL-bax* mRNA was eliminated by the exchange of K-A to D-E, at least in *Se*CspD and *Vc*CspD (Supplemental Fig. S6), consistent with what was seen in *Ec*CspD. Except for *Vc*CspD, the accumulation of *gdx* mRNA was less prominent than the control *Ec*CspA, as with *Ec*CspD; in the *Vc*CspD overproduction strain, while some *gdx* induction was seen, it was slightly reduced compared to *Ec*CspA (Fig. 6C, compare lanes 3–7 with lane 10). The target specificity was evaluated with a calculation of the relative efficiency for *baxL* to *Ec*CspD minus that for *gdx* to *Ec*CspA. The specificities of the tested K-A type proteins strikingly matched the results found with *Ec*CspD (Fig. 6D).

The tested K-E type proteins induced *baxL-bax* mRNA, but less strongly compared to *Ec*CspD (Fig. 6C, compare lanes 8–9 with lane 2), which is consistent with the effect of *Ec*CspD-KE mutation (see Fig. 4B). The *gdx* mRNA was induced by these K-E type proteins at a level similar to that for *Ec*CspA (Fig. 6C, compare lanes 8–9 with lane 10). Reflecting that the relative efficiency for *baxL* to *Ec*CspD was less than that for *gdx* to *Ec*CspA, the difference calculation suggests the specificities of K-E type proteins is similar to that of *Ec*CspA (Fig. 6D).

Overall, the results suggest the usefulness of K-A in the loop region; using these two amino acids, the *Ec*CspD-type target specificity can be distinguished from that of *Ec*CspA and K-E type proteins, and possibly other types of Csps.

### Target recognition by K-E type protein

**FIGURE 7.**
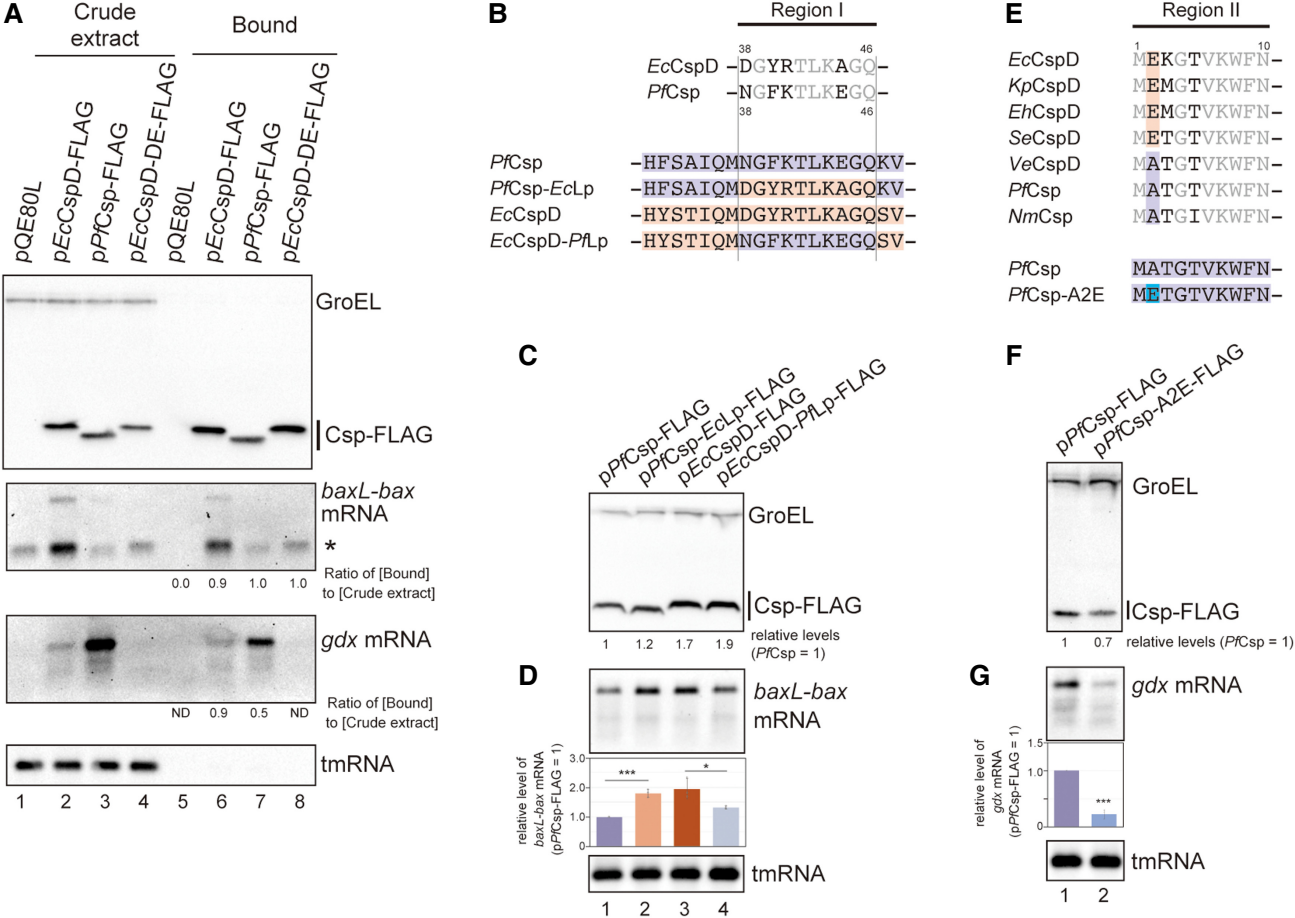
Target recognition by *Pf*Csp. (*A*) In vivo binding of *Pf*Csp-FLAG and *Ec*CspD-DE-FLAG to *baxL-bax* mRNA and *gdx* mRNA. Crude extract was prepared from MG1655 cells harboring the indicated plasmids and subjected to the pull-down assay using anti-FLAG M2 magnetic beads as described in Materials and Methods. The ratio of (Bound) relative to (Crude extract) is shown at the *bottom*; in the calculation for *baxL-bax* mRNA, the sum of the full-length and truncated (*) was used. Data shown are representative of the two independent experiments. (*B*) Sequence alignment of Region I. Conserved residues between *Pf*Csp and *Ec*CspD are shown in gray. The regions originating from *Pf*Csp and *Ec*CspD are marked in purple and orange, respectively, and mutation sites are indicated in the colors of the originating Csp. (*C*,*D*) Expression and properties of mutants. MG1655 cells harboring the indicated plasmids were grown in LB-ampicillin medium. At an OD_600_ of 0.3, 0.2 mM IPTG was added to cultures to induce Csps. Incubation was continued for 30 min. (*C*) The protein sample was subjected to western blotting using anti-FLAG monoclonal antibody and anti-GroEL polyclonal antibodies. (*D*) The RNA sample was subjected to northern blotting using probes for *baxL-bax* mRNA or tmRNA. Quantitation data is shown *below* the northern blot; averages of relative RNA levels were calculated from three independent experiments, with error bars representing the standard deviations. The RNA sample of *Pf*Csp was set to 1. (*E*) Sequence alignment of the N-terminal portion. Conserved residues between the K-A and K-E proteins are shown in gray. *E* and *A* at the second position are marked in orange and purple, respectively. (*F*,*G*) Effect of point mutation in the position 2 of *Pf*Csp on *gdx* induction. MG1655 cells harboring the indicated plasmids were analyzed as in *C*. Relative RNA levels were calculated, with the RNA sample of *Pf*Csp set to 1. Statistical significance was calculated using an unpaired two-tailed Student's *t*-test. (*) *P* < 0.05, (***) *P* < 0.001.

To study the K-E type proteins, the binding property of *Pf*Csp to RNAs was examined. An in vivo pull-down assay with anti-FLAG M2 magnetic beads demonstrated that the *baxL-bax* and *gdx* mRNAs, but not tmRNA, were copurified with *Pf*Csp, and the enrichment ratios for these bands did not correlate with the accumulation levels of these RNAs in extracts (Fig. 7A, lanes 3 and 7). The mutant *Ec*CspD-DE, which lost the activities to both induce *baxL* and attenuate SgrS termination, was similarly found to still bind *baxL-bax* mRNA (Fig. 7A, lanes 4 and 8); *gdx* mRNA was not detected under this assay condition, possibly reflecting the observation that in the CspD-DE overproduction strain the induction level of *gdx* was somewhat reduced compared to the wild-type CspD (see Fig. 3). These suggest that, as discussed above with *Ec*CspD and *Ec*CspA in Figure 1, the differences in the tested Csps reflect differences in the ability to promote induction of these RNA bands rather than that to bind to the RNA targets.

To identify the regions involved in the target recognition and induction by *Pf*Csp, we compared it with *Ec*CspD. A series of chimeric proteins of *Pf*Csp and *Ec*CpsD showed that the chimeras containing Region I of *Ec*CspD induced *baxL-bax* mRNA at comparable levels to *Ec*CspD (Supplemental Fig. S7, lanes 6–7 to lane 2). Consistent with this, the exchange of the loop regions rendered *Pf*Csp active for the *baxL-bax* mRNA and diminished the effect of *Ec*CspD on *baxL* (Fig. 7B–D). The effects of these Cps mutants nicely agree with the results of the CspD-KE mutant (see Fig. 4B), suggesting that the acidic residue E in the loop of *Pf*Csp impedes *baxL* induction.

For *gdx* mRNA, all the chimeras of *Pf*Csp and *Ec*CspD reduced its induction compared to the wild-type *Pf*Csp, and replacement of the N-terminal portion particularly led a more severe reduction of the *gdx* mRNA (Supplemental Fig. S7B, compare lanes 3–5 with 6–8). According to the alignment of Csps, we noticed that K-E type proteins have an alanine residue in the second position (Fig. 7E). This position is dominated by a glutamate residue in *Ec*CspD and three closely related proteins (*Kp*CspD, *Eh*CspD, and *Se*CspD). In contrast, *Vc*CspD, which has an alanine in the second position, induced the *gdx* mRNA to some extent, as K-E type proteins did (see Fig. 6C). These results suggested that E or A at the second position also participates in *gdx* induction, just as *Ec*CspA recognizes the *gdx* mRNA using an N-terminal extension containing a lysine residue. To examine this possibility, a *Pf*Csp mutant in which A was substituted with E was generated (Fig. 7E,F). The *gdx* induction was weakened significantly by the A2E substitution (Fig. 7G), indicating that the N-terminal portion serves a role in the recognition of *gdx* mRNA.

## DISCUSSION

Csps are small RBPs, which are intimately involved in adaptation to environmental changes. In model bacteria such as *E. coli*, there are multiple genes encoding Csps, each of which functions under the condition where it is induced. In addition to the regulation at the expression level, Csps were also shown to individually exhibit inherent target specificity despite their high similarity. In this study, we dissected Csps using two model targets, *baxL-bax* mRNA and *gdx* mRNA. Functional analyses with chimeras and site-directed mutants successfully identified the loop region of *Ec*CspD and the N-terminal portion of both *Ec*CspA and *Pf*Csp as the regions each involved in the target specificity. In these regions, single or a few charged amino acid residue(s) play the important role in the activity for the cognate targets.

### Model of Csp action on a target RNA

In this study, we have demonstrated that acidic residues in the loop diminished the activities of Csps for the *baxL* uORF RNA and an sRNA, SgrS (Figs. 3 and 6), and presumably also for other similar RNA targets. The identity of these loop residues therefore confers target specificity on Csps by interfering with their activities on at least some RNAs. Such an action of acidic residues was indicated in a previous study on RNA chaperone Hfq (Panja et al. 2015). The acidic residues around the RNA-binding surfaces of an Hfq homohexamer secure the accuracy of small RNA regulation through limiting its action on nonspecific RNAs.

**FIGURE 8.**
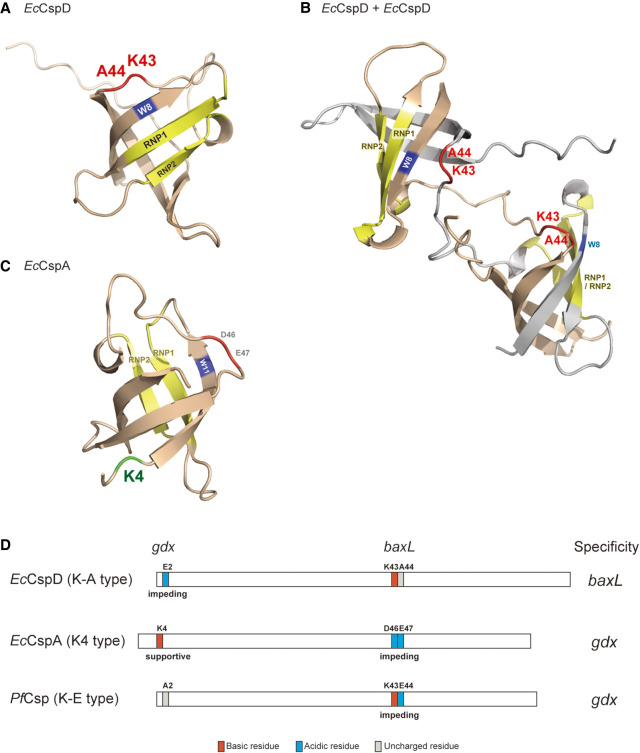
Models of Csp actions and definition of their specificities. (*A*,*B*) Predicted structures of *Ec*CspD monomer (*A*) and homodimer (*B*). The monomer structure was obtained from AlphaFold Protein Structure Database (Jumper et al. 2021; Varadi et al. 2022). The homodimer structure was predicted with AlphaFold-Multimer (Jumper et al. 2021; Evans et al. 2022); one is shown in wheat, and another is in gray. K43 and A44 in the loop are shown in red. RNA-binding motifs, RNP1 and RNP2, and a tryptophan residue in the vicinity are shown in yellow and blue, respectively. (*C*) The structure of *E. coli* CspA monomer (Protein Data Bank ID: 1MJC). K4 in the N-terminal portion is shown in green. (*D*) Cartoon models summarizing the definition of specificities of the tested Csps. The residues identified in this study are highlighted: acidic residues in blue; basic residues in red; uncharged residues in gray.

We constructed structural models of *Ec*CspD to consider how charged residues in the loop affect the actions on targets (Fig. 8). We used alphaFold2 to predict the monomeric as well as dimeric structures of *Ec*CspD as it was reported to form a homodimer (Yamanaka et al. 2001). In the monomer, although a C-terminal extension is uniquely present in *Ec*CspD, the overall predicted structure resembles *Ec*CspA (PDB: 1mjc) (Supplemental Fig. S8A). Based on the monomer structure, K-A residues appear to align with the RNA-binding motifs, RNP1 and RNP2, with flanking W8 (Fig. 8A). This positioned tryptophan residue was shown to be involved in binding to a short RNA in the crystal structure of *B. subtilis* CspB (Sachs et al. 2012), and thus the position of K-A residues may be a location where it can act on a target RNA (Supplemental Fig. S8B). In the dimer structure model, two molecules of *Ec*CspD were predicted to exchange their β-strands, as was observed in the crystal structure of *N. meningitidis* Csp and *Bacillus caldolyticus* CspB (Max et al. 2007; Ren et al. 2008). In this model, the position of K-A is more indicative of their importance because these residues are flanked by two RNA-binding motifs (Fig. 8B). One can envision that if acidic residues are present at the K-A position, these residues negatively act on some target RNAs bound via the two RNA-binding motifs.

Of note, *baxL-bax* mRNA was coprecipitated with *Ec*CspA, *Pf*Csp and a mutant *Ec*CspD-DE, all of which acidic residue(s) are present at the loop position, and SgrS-S was stabilized in the *Ec*CspD-DE overproduction strain (Figs. 1C, 3E, and 7A). These results suggest that acidic residues in the loop do not fully impair the ability to bind the target RNAs but create preferences for some RNAs.

Our results also showed that a K43A replacement in the loop of *Ec*CspD did not reduce the induction of *baxL-bax* mRNA, indicating that the positively charged property of the loop is not necessary for the activity with *baxL* (Fig. 4B). However, given that the lysine residue at this position is well conserved dominantly among K-A and K-E type groups (Fig. 5A), it is possible that this lysine may have a role not detected in our experiments, all of which used overproduction of given Csps.

The N-terminal portion was identified as another recognition site that is needed to recognize the *gdx* riboswitch, and presumably also similar targets. For *Ec*CspA, the lysine residue at position 4 was necessary for *gdx* recognition (Fig. 3H). In addition, a mutant *Pf*Csp-A2E reduced the induction of *gdx* mRNA (Fig. 7F). These results are consistent with the suggestion that the charged property of the N-terminal portion is important for *gdx* recognition. According to the structure of *Ec*CspA (Fig. 8C), the N-terminal portion containing K4 appears to be located at a distance from the RNA-binding motifs, implying that the action of K4 on the *gdx* riboswitch is likely not direct. Note that previous studies with in vitro assays showed that a stable state of *B. subtilis* CspB seems to be marginal and that the thermostability of it was affected by single amino acid substitution at a certain position (Schindler et al. 1995; Perl et al. 2000). Here, we observed that some of the chimeras and mutants did not accumulate to expected levels (Figs. 2C, 3G). Future work should include understanding whether the stability of the native Csps contributes significantly to their functions under various growth conditions.

### How to define the target specificity of the tested Csps

Our results point to components defining the target RNA specificity of the tested Csps (Fig. 8D). For *Ec*CspD, E2 in the N-terminal portion hinders the recognition for *gdx* mRNA while K43-A44 does not reduce the recognition for *baxL*, leading to a preference for the *baxL* compared to *gdx*. For *Ec*CspA, D46 and E47 in the loop interfere with the recognition for *baxL-bax* mRNA, and K4 in the N-terminal portion assists it to recognize *gdx* mRNA, resulting in its preference for *gdx*. For *Pf*Csp, E44 in the loop interferes with the recognition for *baxL-bax* mRNA, whereas the uncharged N-terminal portion does not affect the recognition for *gdx* mRNA. In this case, the specificity directs toward *gdx* mRNA because the negative effect for *baxL* is profound.

It should be noted that we sought to define the region within the RNA necessary for Csp action, using translational *mCherry* or other reporters. However, because *mCherry* and other reporters were themselves affected by overproduction of the Csps, this was not possible. In the future, identifying targets more globally, using RIP-seq or other techniques, combined with dissecting targets of interest in their native context will be needed to fully understand what structures or sequences in the target are critical for specific Csp action.

### Prediction of the target specificity using identified residues

The K-A of the loop is found only in CspD among *E. coli* Csps (Fig. 4C and Supplemental Fig. S3). This prompted us to extend the comparison to Csps in other bacteria. On examination of the amino acid arrangement with a phylogenetic tree, the proteins containing K-A in the loop formed a monophyletic clade (Fig. 5A). Although only a subset of the K-A type proteins had been informatically annotated as CspD, we demonstrated that these proteins indeed have a propensity to recognize similar targets, and therefore a similar specificity to *Ec*CspD (Fig. 6). We conclude that the exploration using K-A of the loop was a highly effective approach for classification of the *Ec*CspD-like proteins. CspD of *K. pneumoniae* was reported to be involved in antibiotic resistance (Ma et al. 2023). Our annotation of CspD-type proteins based on the target specificity may help to elucidate the mechanism of drug resistance in *K. pneumoniae*.

### Biological roles of *Ec*CspD-like K-A type proteins

We began this study to ask how as well as why CspD, unlike other Csps of *E. coli*, acts as an attenuator of transcription termination for SgrS, an Hfq-binding sRNA. Such sRNAs function as posttranscriptional regulators of gene expression, whose roles in response to environmental cues have been established in *E. coli* and other bacteria, particularly for Gamma- and Betaproteobacteria (Holmqvist and Wagner 2017; Hör et al. 2020; Moon et al. 2021; Papenfort and Melamed 2023). In the process of sRNA production, terminating transcription at the proper position is required because the 3′ end is the portion responsible for sRNA binding to its partner, Hfq (Morita et al. 2015, 2017). Therefore, CspD, which is induced under poor growth conditions, has an ability to disturb sRNA regulatory pathways by preventing the formation of the functional 3′ end of sRNAs.

Intriguingly, regulation by Hfq-dependent sRNAs appears to be very rarely employed in *B. subtilis* and some members of Gram-positive bacteria (Watkins and Arya 2023). We noticed that the difference in the contribution of sRNA/Hfq at least between *E. coli* (Gammaproteobacteria) and *B. subtilis* (Bacillota) is apparently consistent with the distribution pattern of K-A type proteins including *Ec*CspD (Fig. 5B and Supplemental Fig. S5A). It is also interesting that, although Betaproteobacteria use the sRNA/Hfq regulation, *Ec*CspD-like K-A type proteins were not found in their genomes. These support the involvement of *Ec*CspD in the sRNA production and raises a possibility that genes encoding K-A type Csps spread together with those encoding Hfq in Gammaproteobacteria, as, while the sRNAs sequences are not widely conserved, the role of Hfq in mediating sRNA function is conserved.

### Summary

This study proposes the definition and molecular basis of the specificity of some Csps, which selectively act on their preferred target RNAs. However, what characteristics of the target RNAs are preferred by each type of Csp remains to be studied. To elucidate this, mutational analysis in the *baxL-bax* and *gdx* mRNA as well as recent RNA-sequencing methods such as RIP-seq are needed. A comprehensive view of Csps as well as their cognate target RNAs will resolve why multiple *csp* genes are present in some bacteria. In addition, Csps are used as material molecules for genetic engineering (Higuchi et al. 2020). We anticipate that further studies on this direction may make a Csp designable to control a given RNA of interest.

## MATERIALS AND METHODS

### Bacterial strains and plasmids

*E. coli* K12 strains and plasmids used in this study are listed in Supplemental Table S2A. MG1655 was used as the parent strain; for experiments in Figure 3D, TM772 (W3110*mlc*^−^ Δ*sgrR-sgrS* Δ*hfq*::*cat*) was used. The *cspD-FLAG-cat* allele was constructed with NM1100 (Luo et al. 2023), according to the modified Datsenko–Wanner protocol, using pSU313 harboring the *FLAG-cat* sequence (Uzzau et al. 2001). The allele was moved into MG1655 by P1 transduction, and the *cat* gene was removed using plasmid pCP20 (Cherepanov and Wackernagel 1995).

Plasmids of pQE-Csp and their derivatives were constructed as follows. Inserts were amplified from the genomic DNA of MG1655 or the template plasmid; or a synthetic gene fragment (gBlock, IDT) was used as an insert. The template DNA corresponding to each plasmid and the respective oligonucleotide combinations are listed in Supplemental Table S2A. Sequences of oligonucleotides and gBlock fragments are listed in Supplemental Table S2B. In the construction of chimeras and site-directed mutants, DNA fragments containing the mutation site were created by overlapping PCRs from upstream and downstream from the site (Morita and Aiba 2019). The resulting product was digested with EcoRI and HindIII, then cloned into pQE80L. Construction of pCspF-FLAG and pCspH-FLAG was designed to replace the initiation codon of UUG with AUG. For the construction of pCspG-FLAG, a gBlock gene fragment was used as the insert, in which the EcoRI site in the coding sequence (CDS) was mutated by synonymous substitution. For pQE plasmids carrying a *csp* gene from other bacteria, gBlock gene fragments were digested with EcoRI and HindIII, then cloned into pQE80L. All plasmid constructs were verified by Sanger sequencing of the inserted region.

### Growth conditions

Cells carrying the indicated plasmids were grown at 37°C in LB medium supplemented with ampicillin (100 μg mL^−1^) when necessary. In Figure 3D, we used LB medium supplemented with ampicillin (50 μg mL^−1^) and kanamycin (15 μg mL^−1^). Overnight cultures were diluted 100-fold into the same fresh medium. Cell growth was monitored by determining optical density (OD_600_).

### Western blotting

Cells were harvested from cultures (500 μL) by centrifugation, and the cell pellets were suspended with Laemmli Sample Buffer (BioRad) including 5% 2-mercaptoethanol. One microliter of the pellet suspension by sample buffer equivalent to the value of an OD_600_×100 µL was defined as 1 unit; 0.4 unit of protein sample was used. The sample was resolved on 16.5% SDS-PAGE, then transferred onto a PVDF membrane (BioRad). The membrane was treated with an anti-FLAG monoclonal antibody (Wako #018-22381) and anti-GroEL antibody produced in rabbit (Merck, Sigma-Aldrich #G6532). Anti-Mouse IgG and Anti-Rabbit IgG (Cytiva NA931 and NA934) were used as peroxidase-linked secondary antibodies. Signals were visualized by the Lumi-Light Western Blotting Substrate (Merck), then captured using the imaging system ChimiDoc XRS Plus (BioRad). All Blue Prestained Protein Standards (BioRad) was used as protein size markers.

### Northern blotting

Total RNAs were isolated as described in Aiba et al. (1981). To detect *baxL-bax* mRNA, *gdx* mRNA, or tmRNA, 3 μg, 3 μg, or 1 μg of RNA sample was resolved by 1.7% agarose gel electrophoresis in the presence of formaldehyde. To detect SgrS-S, 5 μg of RNA sample was resolved by 10% polyacrylamide gel electrophoresis in the presence of 7 M urea. RNAs in the gel were blotted onto a nylon membrane, positively charged (Roche). The RNAs were visualized by using a detection system with digoxigenin (DIG) (Roche), then captured and quantified using the imaging system ChimiDoc XRS Plus (BioRad). The following RNA probes were prepared by the DIG RNA Labeling Kit (Roche); the antisense corresponding to the −63 to +137 region relative to the AUG start codon of *bax* (*baxL-bax* probe); the antisense corresponding to the −81 to +119 region relative to the AUG start codon of *gdx* (*gdx* probe); the antisense corresponding to the +168 to +198 portion of *sgrS* (SgrS-S probe). The tmRNA probe of a 363-bp DNA fragment was prepared by PCR using DIG-dUTP. Prestain Marker for RNA High (BioDynamics Laboratory, Inc.) and DIG Labeled Blue Color Marker for Small RNA (BioDynamics Laboratory, Inc.) were used as RNA size markers for agarose gel electrophoresis and polyacrylamide gel electrophoresis, respectively.

### Pull-down assay

Cells were grown in 20 mL of LB medium at 37°C. At OD_600_ = 0.3, 0.2 mM IPTG was added, and incubation was continued for 30 min. Cells were harvested and washed with 10 mL STE buffer (100 mM NaCl, 10 mM Tris-HCl at pH 8.0, and 1 mM EDTA). The cell pellet was suspended in 1 mL IP buffer (20 mM Tris-HCl at pH 8.0, 0.2 M KCl, 5 mM MgCl_2_, 10% glycerol, and 0.1% Tween20). The cell suspension was crushed by mT-01 Beads Crusher (Taitec) with ϕ0.350–0.500 mm of glass beads. After centrifugation at 12,000*g* for 3 min at 4°C, the supernatant (crude extract) was incubated with 10 μL of anti-FLAG M2-magnetic beads suspension (Millipore) in 460 μL of IP buffer for 20 min at 4°C. The magnetic beads were collected by magnet and washed twice with 0.5 mL of IP buffer. The proteins bound to the beads were eluted with 50 μL of IP buffer containing 0.4 mg mL^−1^ FLAG peptide (Sigma-Aldrich) and used as bound fraction. To analyze proteins, crude extract (0.25 μL) and bound fraction (0.25 μL) were subjected to western blotting. To analyze RNAs, crude extract (9 μL, 9 μL, and 3 μL) and bound fraction (9 μL, 9 μL, and 3 μL) for *baxL-bax* mRNA, *gdx* mRNA, and tmRNA, respectively, were treated with phenol, precipitated, and washed with ethanol. Each precipitant was dissolved in RNA buffer (0.02 M sodium acetate at pH 5.2, 0.5% SDS, and 1 mM EDTA). The RNA samples were subjected to northern blotting.

### Bioinformatic analysis on Csp phylogeny

A total of 14,363,661 CDSs from 4032 bacterial genomes were downloaded in GeneBank format from the NCBI RefSeq database (O'Leary et al. 2016) on October 27, 2022. The members of Csp were collected by PSI-BLAST (Altschul et al. 1997) version 2.5.0+ (12 iterations, E-value of ≤1×10^−3^) with 12 query amino acid sequences: a set of nine *E. coli* Csps (GCF_000005845.2: NP418012.1, NP416075.1, NP416337.1, NP415401.1, NP415156.1, NP416076.1, NP415510.1, NP415509.1, NP416070.1), *S. enterica* CspC and CspE (GCF_000210855.2: CBW17860.1, CBW16717.1), and *B. subtilis* CspB (GCF_000009045.1: NP388791.1). In total, 2440 CDSs were obtained from a total of 1019 genomes, and their amino acid sequences were aligned with MAFFT (version 7.490) with L-INS-i option (Katoh and Standley 2013). Gap removal was performed using trimAI (version 1.4, rev15) (Capella-Gutiérrez et al. 2009). Jalview (version 2.11.2.6) (Waterhouse et al. 2009) was used to visualize multiple sequence alignment, and the sharing of RNA-binding motifs among the Csp members was confirmed.

For the construction of a phylogenetic tree of Csps, duplicate identical CDSs were excluded by using CD-HIT (version 4.8.1) (Fu et al. 2012), resulting in 1573 CDSs. IQ-TREE (version 2.0.3) (Minh et al. 2020) was used to construct the phylogenetic tree of Csps. ModelFinder (Kalyaanamoorthy et al. 2017) was used to find the best-fit substitution model, resulting in the selection of LG + R10. Additionally, an ultrafast bootstrap approximation (UFBoot) (Hoang et al. 2018) was used to overcome the computational burden required by the nonparametric bootstrap. iTOL (version 6.7.2) was used to visualize the phylogenetic tree of Csps, and the Csps containing a specific amino acid residue at a certain position were color-coded. Python scripts for analyses are available on GitHub (https://github.com/seanhasegawa/CSP_2024).

### Distribution of Csps in bacteria

For the construction of a phylogenetic tree that is composed of 4032 or 1019 bacterial genomes, the nucleotide sequences of 16S rRNA were aligned using MAFFT (version 7.490) and trimAI (version 1.4. rev15). IQ-TREE (version 2.0.3), ModelFinder and UFBoot were used to construct the phylogenetic trees. The phylogenetic trees were visualized on iTOL (version 6.7.2). Of 2440 CDSs, the total number of the included Csps was mapped to the tree of 4032 bacteria, and those classified by identified residues were mapped to the tree of 564 bacteria. Python scripts for analyses are available on GitHub (https://github.com/seanhasegawa/CSP_2024).

Two-sample nonparametric tests were employed to examine the genomic characteristics of bacterial genomes having one or two Csps (OneOrTwoCsps, *n* = 683) compared to those having three or more Csps (ThreeOrMoreCsps, *n* = 336). In analyzing the relationship between the number of Csps included and size of the host genome or its GC content, IBM SPSS Statistics (version 29.0.1.1) was used to evaluate significant differences within each pair of groups. To determine the appropriate statistical test, normality tests were conducted on the data sets. The Kolmogorov–Smirnov and Shapiro–Wilk tests were applied, both of which yielded *P*-values <0.001 for the genome size and GC content of OneOrTwoCsps and ThreeOrMoreCsps. The hypothesis that these data follow a normal distribution was rejected, leading to the conclusion that the data are not normally distributed. Based on the absence of normal distribution, we employed the Mann–Whitney *U* test, a nonparametric two-sample test that does not assume normality in the data sets.

### Structure prediction of *E. coli* CspD

A predicted monomer structure of *Ec*CspD was downloaded from AlphaFold Protein Structure Database (Jumper et al. 2021; Varadi et al. 2022). To predict a homodimer structure of *Ec*CspD, AlphaFold multimer (version 2.3.1) (Jumper et al. 2021; Evans et al. 2022) was used. The prediction was run with all the genetic databases available on AlphaFold2, and the best ranked CspD homodimer was selected. Both predicted and known structures were visualized by PyMOL (The PyMOL Molecular Graphics System, Version 2.5.0, Schrödinger, LLC) to form the model shown in Figure 8 and Supplemental Figure S8.

## DATA DEPOSITION

Python scripts to analyze phylogeny and distribution of Csps are available at https://github.com/seanhasegawa/CSP_2024. The data that support the findings in this study are available from the corresponding author upon request.

## SUPPLEMENTAL MATERIAL

Supplemental material is available for this article.

## Figures and Tables

**Satoshi Hasegawa:**
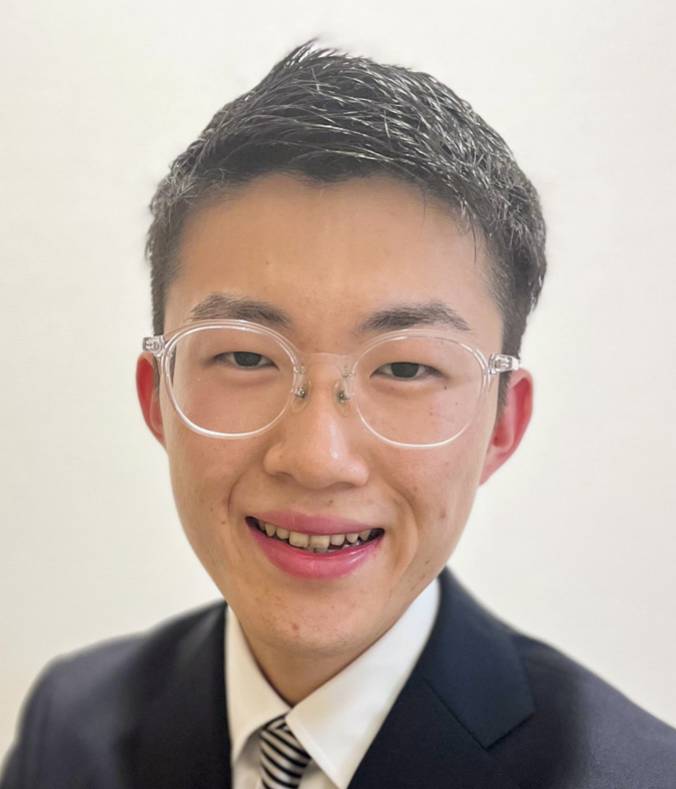


## MEET THE FIRST AUTHOR

***Meet the First Author(s)* is an editorial feature within *RNA*, in which the first author(s) of research-based papers in each issue have the opportunity to introduce themselves and their work to readers of *RNA* and the RNA research community. Satoshi Hasegawa is the co-first author of the paper, “An internal loop region is responsible for inherent target specificity of bacterial cold-shock proteins,” along with Rerina Inose. Satoshi is a fourth-year undergraduate student in Dr. Teppei Morita's laboratory at the Institute for Advanced Biosciences, Keio University, Japan. His research focuses on the molecular mechanisms of transcription elongation across regulatory RNA elements, specifically mediated by cold-shock proteins (Csps).**

**What are the major results described in your paper and how do they impact this branch of the field?**

The major results of our paper provide a molecular basis for the selective recognition of RNA targets by Csps, which share a protein fold for the cold-shock domain that contains RNA-binding motifs and play biological roles in fundamental processes. We identified that two amino acid residues K43-A44 in *E. coli* CspD are responsible for determining the target specificity, whereas the N-terminal portion containing K4 of *E. coli* CspA is important for the specific target recognition. A phylogenetic tree of Csps suggested that these identified critical residues can be effective for the subclassification of bacterial Csps. Using the results of mutation analyses in conjunction with predicted structural models, we discussed how the tested Csps act selectively on their cognate target RNAs. Our study sheds light on a new aspect of the functional specialization of an important and widespread RNA-binding protein family.

**What led you to study RNA or this aspect of RNA science?**

I was drawn to RNA science by the way organisms adapt to environmental changes through regulation of gene expression. Even in unicellular organisms, this complex regulation allows them to process vast amounts of genomic information, selecting only what is needed at any given time. I was particularly amazed by how such intricate information processing happens in cells so small, and this inspired me to dive deeper into understanding the mechanisms.

**Are there specific individuals or groups who have influenced your philosophy or approach to science?**

Professor Masaru Tomita, the founder of our research institute, had a profound impact on my approach to science. In his class, *Evolution of Life and Intelligence*, he shared a statement that left a lasting impression: Humans, with all their intelligence, creativity, and complexity, are built on just 1 GB of genetic information. I was amazed by the idea that something as remarkable and resilient as a human being could arise from such a small amount of data, especially when a laptop's operating system requires at least 10 GB. His insight inspired me to delve deeper into understanding the fundamental mechanisms of life.

**What are your subsequent near- or long-term career plans?**

I plan to continue my research by first pursuing a master's degree, where I will explore in more detail how Csps interact with target RNAs. After that, I hope to pursue a PhD abroad, giving me the chance to collaborate with leading PIs in molecular biology. In the long term, I aim to contribute to advancements in biotechnology, whether in academia or industry.

**What were the strongest aspects of your collaboration as co-first authors?**

The strongest aspect of our collaboration as co-first authors was the complementary nature of our skills. Rerina Inose is a skilled lab technician, particularly in wet lab techniques like northern blotting and plasmid construction. Her precision and consistency in these experiments were crucial for the functional analyses of Csps. Moreover, it wasn't just the two of us; through continuous discussions with Dr. Morita and our co-authors, we were able to reach the conclusions presented in our study.
